# Lethal systemic and brain infection caused by *Prototheca zopfii* algae in a patient with acute myeloid leukemia

**DOI:** 10.1016/j.mmcr.2021.01.004

**Published:** 2021-01-28

**Authors:** Stephanie Herold, Tristan Klodt, Daniela Toelle, Martin Dennebaum, Elena Lippe, Cleo Walz, Joachim Kaes, Andreas Kreft, Ekkehard Siegel, Markus Munder, Daniel Teschner

**Affiliations:** a Department of Hematology, Medical Oncology, and Pneumology, University Medical Center of the Johannes Gutenberg University, Langenbeckstr. 1, 55131, Mainz, Germany; b Institute of Neuropathology, University Medical Center of the Johannes Gutenberg University, Langenbeckstr. 1, 55131, Mainz, Germany; c Institute of Clinical Microbiology and Hygiene, University Medical Center of the Johannes Gutenberg University, Langenbeckstr. 1, 55131, Mainz, Germany; d Institute of Pathology, University Medical Center of the Johannes Gutenberg University, Langenbeckstr. 1, 55131, Mainz, Germany; e Institute of Forensic Medicine, University Medical Center of the Johannes Gutenberg University, Langenbeckstr. 1, 55131, Mainz, Germany; f Department of Cardiology, University Medical Center of the Johannes Gutenberg University, Langenbeckstr. 1, 55131, Mainz, Germany

**Keywords:** *Prototheca zopfii*, Protothecosis, Encephalitis, Leukemia, Immunodeficiency

## Abstract

Systemic protothecosis is an exceptionally rare cause of sepsis with few available therapeutic options. Here, we report on a female patient with newly diagnosed acute myeloid leukemia who died after start of chemotherapy due to a severe septic shock caused by a disseminated systemic infection with *Prototheca zopfii* including encephalitis.

## Highlights

• Prototheca species are the only achlorophyllic algae known to cause infections in humans.
• Systemic protothecosis is usually associated with an underlying immunodeficiency.
• We report the first human case of *P. zopfii* brain infection in Germany.

## 1 Introduction

Protothecosis is a rare disease in humans, and two third of protothecosis cases are localized infections with cutaneous lesions. However, when presenting as a disseminated protothecosis this is usually associated with an underlying immunodeficiency [1,2].

*Prototheca* spp. are achlorophyllic unicellular organisms with 3–30 μm in diameter and belong to the Chlorophyceae. They can be found ubiquitously in nature [3] and are primarily described in literature as a cause for bovine mastitis [4]. Within the *Prototheca* species, the most common human pathogens are *P. wickerhamii* and to a lesser extent *P. zopfii* [2,5]. The first documented infection in humans was reported in 1964 in a rice farmer having a cutaneous infection of the lower limb [6]. For unknown reasons, protothecosis also frequently occurs as olecranon bursitis (15–25% of published cases) [5,7]. In systemic protothecosis, half of the documented cases were reported in immunodeficient patients [7]. In cancer patients suffering from protothecosis, hematological malignancies are clearly overrepresented (7 of 13 patients) as depicted in a review of Torres et al. [5]. In case of disseminated infection, Amphotericin B was reported to be the most effective therapeutic agent [[7], [8], [9]].

## 2 Case

In February 2018, a 61-year-old female patient was admitted to our hospital because of a newly diagnosed acute myeloid leukemia (AML). Peripheral blood showed pancytopenia with no laboratory signs of systemic infection or organ dysfunction (day 0). She presented with a good performance status (ECOG 1), and obesity grade III. Prior to chemotherapy, transthoracic echocardiography showed a good heart function but lung function testing revealed a restrictive ventilation deficit.

Formerly, the patient was diagnosed with breast cancer in 2014 and was successfully treated by surgery and radiochemotherapy. In 2015, she had experienced a putrid diverticulitis and had undergone sigmoid resection. Additionally, homozygous haemochromatosis regularly treated by phlebotomy was reported. Furthermore, we newly diagnosed diabetes mellitus type 2 on admission and treated it with insulin according to our institutional guidelines.

The patient was scheduled for standard induction chemotherapy with cytarabine and idarubicine (day 1–7). Posaconazole antifungal prophylaxis was initiated according to our institutional guidelines. On day 6, fever and diarrhea occurred and empirical antibiotic treatment with piperacillin/tazobactam was started. Subsequently, *Klebsiella pneumoniae* and *Enterococcus faecium* were detected in blood cultures and the antibiotics were switched to meropenem and teicoplanin based on bacterial in vitro susceptibility testing. The patient required additional oxygen supply on day 8, and consequently a chest CT-scan was performed showing no signs of pneumonia. The central venous catheter was replaced on day 13 to address a suspected catheter-related infection. The patient presented with clinical signs of a paralytic ileus confirmed by abdominal CT scan. Additional transthoracic echocardiography revealed no pathological findings.

**Fig. 1:**
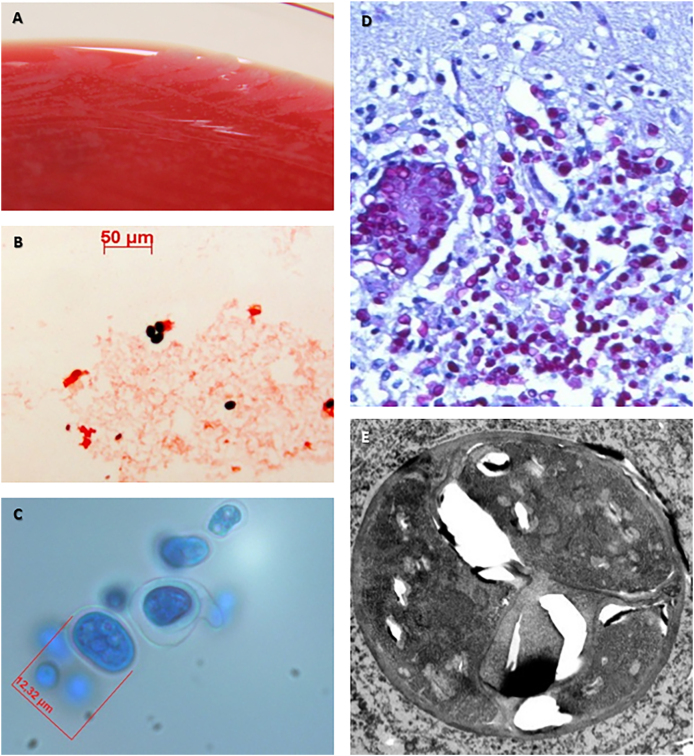
Characterization of *Prototheca zopfii* as relevant infectious agent. *P. zopfii* grew on Columbia blood agar after 24 hours of incubation in form of yeast-like colonies (A). Blood cultures were investigated microscopically. In gram staining, the algae appeared as gram positive (100x; B). In Lactophenol cotton blue staining, unicellular organisms with 10–15μm in diameter and with asymmetrical morula-like structures representing the endospores appeared (400x; C). Microscopically, cerebral cortex displayed multiple round organisms (PAS staining; 400x; D). Ultrastructure of single *P. zopfii* cells was examined in detail using transmission electron microscopy (5000x; E). (For interpretation of the references to colour in this figure legend, the reader is referred to the Web version of this article.)

From day 14 onward, the patient became delirious and a cranial CT scan was performed revealing no signs of intracranial pathology. Reconstitution of the white blood cells with neutrophil granulocytes >500/μL was noted on day 27. Subsequently, the patient started to have a respiratory distress syndrome. As the blood gas analysis unveiled increasing hypercapnia, the patient was transmitted to the intensive care unit. Here, the patient was initially treated with non-invasive ventilation but had to be intubated and mechanically ventilated due to physical exhaustion on day 28. Additionally, catecholamine treatment had to be started due to evolving septic shock, and hemodialysis was initiated due to acute kidney failure and lactate acidosis. On day 25, aerobic blood cultures taken on day 22 showed growth of small, dry, opaque, white, and yeast-like colonies on Columbia agar after incubation at 37 °C for 24 h (Fig. 1, A). Microscopic investigation showed a rather ambiguous and perplexing picture which did not correspond to any conventionally known organism (Fig. 1, B). In lactophenol staining, unicellular organisms with asymmetrical morula-like structures appeared (Fig. 1, C). After genome analysis by molecular biology the pathogen could be identified as green algae named *Prototheca zopfii* on day 31, which was later on confirmed as genotype 2 by additional molecular testing (16S-rRNA sequence analysis). Bronchoalveolar lavage fluid was found to be positive for *P. zopfii* algae one day later. On day 31, liposomal Amphotericin B treatment was initiated at a dose of 5mg/kg body weight. However, the patient deteriorated rapidly, and died on day 32 because of sepsis-induced multi-organ failure.

**Fig. 2:**
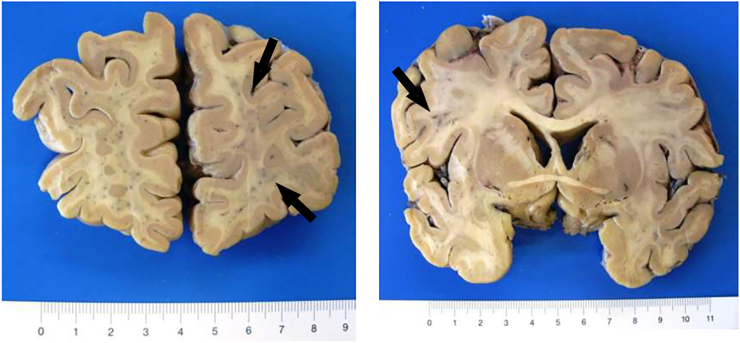
Gross histopathology of the brain (autopsy). Brain tissue showed spotty pattern due to multiple circumscribed white-yellowish lesions (arrows).

The autopsy revealed signs of septic shock and a spotty pattern of the myocardial and renal cutting surfaces due to multiple circumscribed white-yellowish lesions. Histologically, these lesions contained unicellular, round to ovoid organisms with foamy basophilic cytoplasm and a periodic acid-Schiff (PAS) positive cell wall confirming the previously diagnosed protothecosis (Fig. 1, D). Single cells were examined in detail using transmission electron microscopy (Fig. 1, E). The algae were accompanied by dense infiltrates of inflammatory leukocytes. Additionally, algae lesions were found in all five pulmonary lobes, the liver, and in multiple brain loci (Fig. 2), including cerebral cortex, white matter, basal ganglia, and brain stem. During autopsy, 2 samples from each kidney, and 3 samples from the heart were taken and further examined by culture in our microbiological institute. All cultures showed growth of *P. zopfii.* In consequence, the autopsy revealed wide-spread dissemination of *P. zopfii* and confirmed the systemic protothecosis as cause of death.

## 3 Discussion

To the best of our knowledge, this is the first reported human case in Germany of disseminated *P. zopfii* infection affecting the central nervous system (CNS). Recently, two cases of human encephalitis due to *P. zopfii* in immunocompetent patients in Switzerland were published [10]. Interestingly, unlike our patient, those two patients showed no clinical signs of immunodeficiency. In contrast with our own microscopic findings, their brain biopsies revealed a granulomatous pattern with epithelioid/giant cells. Various previous reports of CNS protothecosis in animals also described granulomatous changes [11]. Thus, type and severity of human encephalitis might depend on the cellular immune status. Following this assumption, granulomatous changes might be a morphological correlate for a sufficient host cellular immune defense against *P. zopfii*. Another case report described a disseminated *P. zopfii* infection in a liver transplant patient with severe neurological impairments such as hemiparesis and cerebral magnetic resonance imaging with multiple enhancing lesions suggestive for CNS involvement [12]. No autopsy findings or histological analyses were reported.

Our patient had various risk factors for an opportunistic infection, specifically: AML as a hematological malignancy, diabetes mellitus, metabolic syndrome, a history of breast cancer and a prior gut operation, as well as homozygous hemochromatosis. In systemic protothecosis, hematological malignancies and diabetes were described as associated risk factors [5,13]. The patient initially suffered from neutropenic enteritis and a disturbed mucosal barrier was therefore present. In line with current guidelines, we administered posaconazole prophylaxis. However, posaconazole is not reported to be effective in preventing protothecosis.

Despite intense diagnostic workup, we were not able to detect the source of infection with *P. zopfii*. In general, the entry route of *P. zopfii* into the human organism is controversial. Some studies suggest traumatic inoculation [3,5]. The presented patterns of multiple intra- and extracerebral algae lesions support the hypothesis of hematogenous dissemination indicating the potential of *P. zopfii* to cross the blood-brain barrier.

As *Prototheca* is the only achlorophyllic algae known to cause very rare infections in humans, disseminated protothecosis is not usually suspected to be the cause of sepsis [5,7]. Microscopically, the propagated cultures did not show typically fungal or bacterial structures and thus could easily be interpreted as artefacts. Hence, this constellation connotes a delayed medical diagnosis. Even more crucial are the missing options for targeted therapeutic measures. Since algae are not “designated” as pathogens in human medicine, the substitute therapy with antifungals can only be viewed as makeshift. Existing recommendations are only based on retrospective case reports and on in vitro susceptibility testing [8]. This delay in diagnosis and lack of evidence in treatment guidelines explain the generally poor prognosis of systemic protothecosis with a mortality of up to 52% [5].

This case report of systemic protothecosis demonstrates that physicians should consider uncommon causes of sepsis in patients with antibiotic-refractory fever. We provide this case report to raise awareness towards protothecosis in immunocompromised patients.

## Funding

This research did not receive any specific grant from funding agencies in the public, commercial, or not-for-profit sectors.

## Declaration of competing interest

D.T. received honoraria from ACI, Gilead, iQone, MSD, Novartis, Pfizer and Shire is consultant of advisory board for Gilead, iQone, MSD, and Pfizer and received travel support by Abbvie, Astellas, Celgene, Gilead, Jazz, Medac, and MSD, outside the submitted work. The other author(s) declare no competing interests.
